# Fractalkine receptor (CX_3_CR1) deficiency sensitizes mice to the behavioral changes induced by lipopolysaccharide

**DOI:** 10.1186/1742-2094-7-93

**Published:** 2010-12-17

**Authors:** Angela W Corona, Yan Huang, Jason C O'Connor, Robert Dantzer, Keith W Kelley, Phillip G Popovich, Jonathan P Godbout

**Affiliations:** 1 Department of Molecular Virology, Immunology and Medical Genetics, The Ohio State University, 333 W. 10th Ave, Columbus, OH 43210, USA; 2 Department of Animal Science, University of Illinois, 1201 W. Gregory Drive, 250B Edward R. Madigan Laboratory, Urbana, IL 61820, USA; 3 Institute for Behavioral Medicine Research, The Ohio State University, 460 Medical Center Dr., Columbus, OH 43210, USA; 4 Center for Brain and Spinal Cord Repair, The Ohio State University, 460 W. 12th Ave, Columbus, OH 43210, USA; 5 Department of Neuroscience, The Ohio State University, 333 W. 10th Ave, Columbus, OH 43210, USA; 6 Department of Pharmacology, University of Texas Health Science Center, 7703 Floyd Curl Dr., San Antonio, TX 78229, USA

## Abstract

**Background:**

Interactions between fractalkine (CX_3_CL1) and fractalkine receptor (CX_3_CR1) regulate microglial activation in the CNS. Recent findings indicate that age-associated impairments in CX_3_CL1 and CX_3_CR1 are directly associated with exaggerated microglial activation and an impaired recovery from sickness behavior after peripheral injection of lipopolysaccharide (LPS). Therefore, the purpose of this study was to determine the extent to which an acute LPS injection causes amplified and prolonged microglial activation and behavioral deficits in CX_3_CR1-deficient mice (CX_3_CR1^-/-^).

**Methods:**

CX_3_CR1^-/- ^mice or control heterozygote mice (CX_3_CR1^+/-^) were injected with LPS (0.5 mg/kg i.p.) or saline and behavior (i.e., sickness and depression-like behavior), microglial activation, and markers of tryptophan metabolism were determined. All data were analyzed using Statistical Analysis Systems General Linear Model procedures and were subjected to one-, two-, or three-way ANOVA to determine significant main effects and interactions.

**Results:**

LPS injection caused a prolonged duration of social withdrawal in CX_3_CR1^-/- ^mice compared to control mice. This extended social withdrawal was associated with enhanced mRNA expression of IL-1β, indolamine 2,3-dioxygenase (IDO) and kynurenine monooxygenase (KMO) in microglia 4 h after LPS. Moreover, elevated expression of IL-1β and CD14 was still detected in microglia of CX_3_CR1^-/- ^mice 24 h after LPS. There was also increased turnover of tryptophan, serotonin, and dopamine in the brain 24 h after LPS, but these increases were independent of CX_3_CR1 expression. When submitted to the tail suspension test 48 and 72 h after LPS, an increased duration of immobility was evident only in CX_3_CR1^-/- ^mice. This depression-like behavior in CX_3_CR1^-/- ^mice was associated with a persistent activated microglial phenotype in the hippocampus and prefrontal cortex.

**Conclusions:**

Taken together, these data indicate that a deficiency of CX_3_CR1 is permissive to protracted microglial activation and prolonged behavioral alterations in response to transient activation of the innate immune system.

## Background

Microglia are myeloid derived cells that play a key role in immune surveillance of the central nervous system (CNS) [1,2]. Together with brain macrophages these cells interpret and propagate inflammatory signals in response to activation of the peripheral immune system [3]. Activated microglia produce pro-inflammatory cytokines and secondary messengers which elicit a sickness behavior syndrome [4]. While this sickness response is normally adaptive, amplified or prolonged microglial activation is associated with a myriad of behavioral and cognitive complications [5]. Therefore, tight regulation of microglial activation is necessary to limit cytokine production and maintain a transient neuroinflammatory response.

One mechanism of microglial regulation involves interactions between fractalkine (CX_3_CL1) and fractalkine receptors (CX_3_CR1) [6,7]. Complementary expression of CX_3_CL1 on neurons and CX_3_CR1 on microglia [6-9] establishes a unique communication system whereby neurons constitutively express and release CX_3_CL1 to regulate activation of microglia [10]. For example, pretreatment with neutralizing anti-CX_3_CL1 antibody exaggerates TNFα and 8-isoprostane production after intracerebroventricular (i.c.v.) injection of lipopolysaccharide (LPS) [11]. In cultured microglia or mixed glial cultures, soluble CX_3_CL1 attenuates LPS-induced production of TNFα, IL-6, and IL-1β [12,13]. Moreover, CX_3_CR1-deficiency amplifies microglial IL-1β expression and neurotoxicity in CX_3_CR1^-/- ^mice compared to CX_3_CR1^+/- ^mice after repeated i.p. injections of lipopolysaccharide (LPS) [10].

Recent work using rodent models of aging also support the premise that impaired CX_3_CL1/CX_3_CR1 interactions cause increased microglial activation. For example, CX_3_CL1 mRNA and protein are decreased in the brain of aged rats [14,15] and mice [5]. This age-associated increase in activated (e.g., MHC II^+^) microglia was attenuated following a single i.c.v. injection of CX_3_CL1 [15]. In a related experiment, infusion of a neutralizing antibody to CX_3_CR1 increased the number of MHC II^+ ^microglia in young rats [14]. Along with reductions in brain levels of CX_3_CL1, regulation of CX_3_CR1 expression on the surface of microglia may also be impaired in the aged brain. In comparisons between adult and aged mice, CX_3_CR1 surface expression was decreased on microglia after peripheral injection of LPS. In aged mice, however, the LPS-induced downregulation of CX_3_CR1 on microglia was protracted and corresponded with amplified IL-1β levels in microglia and impaired recovery from sickness behavior [5]. Collectively, these data indicate that CX_3_CL1-CX_3_CR1 interactions are important in normal regulation of microglia and become dysregulated with age.

Clinical and experimental data indicate that there is a cause/effect relationship between inflammation and depression [16,17]. In animal models, proinflammatory cytokine production (e.g., IL-1β, IL-6, or TNFα) following systemic LPS challenge [18-20], chronic infection with Bacillus Calmette-Guerin (BCG) [21,22], stroke [23], or psychological stress [24-26] cause depression-like behavior. One potential mechanism by which inflammatory cytokines promote depression-like behavior is through the activation of the tryptophan degrading enzyme indoleamine 2,3-dioxygenase (IDO) [21,27]. Active IDO converts tryptophan (TRP) into kynurenine (KYN). In microglia [28], KYN is processed by kynurenine monooxygenase (KMO), which produces two highly neuroactive mediators, 3-hydroxykynurenine (3HK) and quinolinic acid (QUIN) [29]. IDO activity and downstream processing of KYN have been shown to cause inflammatory-associated depression in rodent models [20,22]. It is hypothesized that IDO activation is a key mechanism underlying mood and depressive complications because it alters serotonergic, dopaminergic, and noradrenergic neurotransmission [4,17,30,31].

Because CX_3_CL1 and CX_3_CR1 interactions are impaired in the brain of aged rodents [5], we hypothesize that a loss of CX_3_CR1 may be permissive to exaggerated microglial activation and the promotion of a maladaptive sickness response after an innate immune challenge. Therefore, the purpose of this study was to determine the extent to which an acute LPS injection causes amplified and prolonged microglial activation and behavioral deficits in CX_3_CR1-deficient mice (CX_3_CR1^-/-^). Here we show that CX_3_CR1^-/- ^mice exhibited prolonged social withdrawal and depression-like behavior after LPS injection. These behavioral alterations were associated with amplified and prolonged microglial activation.

## Methods

### Animals

Adult (3-6 m) heterozygotes (CX_3_CR1^+/-^) and homozygotes (CX_3_CR1^-/-^) from our in-house specific pathogen free colony were used. These CX_3_CR1 transgenic mice were created in the background strain of C57BL/6 [32]. An EGFP cassette was inserted in place of the first 390 bp of the second exon of the gene for CX_3_CR1 that disrupts the CX_3_CR1 gene and labels all cells that would normally express CX_3_CR1 with a green fluorescent protein [32]. Mice were housed in polypropylene cages and maintained at 25°C under a 12 h light/12 h dark cycle with *ad libitum *access to water and rodent chow. All procedures were in accordance with the National Institute of Health Guidelines for the Care and Use of Laboratory Animals and were approved by The Ohio State University Institutional Laboratory Animal Care and Use Committee.

### Behavior

Locomotor activity, social exploratory behavior, and resignation behavior were determined as previously described [19,20,33]. In brief, mice were acclimated to routine handling 5 days before experimentation. Mice were injected with 0.5 mg/kg LPS and behavioral testing was assayed at the indicated times after injection. Behavioral tests were conducted during the dark phase (between 1200 and 2400) of the photoperiod under infrared lighting. Behavior was scored by a trained observer who was blind to the experimental treatments.

*For locomotor activity*, mice were maintained in their home cage with a floor area of 26 × 20 cm, and activity was video recorded for 3 minutes. On the video records, cages were divided into 6 identical virtual rectangles and the number of line crossings was determined.

*For social exploratory behavior*, a novel juvenile was introduced into the test subject's home cage for a 10-min period. Behavior was videotaped and the cumulative amount of time the adult mouse engaged in social investigation was determined.

*For depression-like behavior*, tail suspension test was performed (TST). Mice were suspended by their tail in a 32 × 33 × 33 cm box and the duration of immobility was determined over a 10 min period. In these experiments, the mice were allowed to acclimate to the testing parameters for 2 minutes prior to scoring the results. Results are expressed as the total immobility for the last 8 minutes of the test.

### IL-1β and CX_3_CL1 protein levels

IL-1β and CX_3_CL1 protein were determined in brain and plasma samples as previously described [5,33]. In brief, mice were anesthetized by CO_2 _inhalation and blood was collected by cardiac puncture. Blood was centrifuged (4000 × g for 15 min at 4°C) and plasma was collected and stored frozen (-80°C) until assaying. Brain samples were homogenized in *lysis buffer *(50 mM Tris-HCl pH 7.4, containing 10% glycerol, 1.0% Triton-X 100, 100 mM NaCl, 50 mM NaF, 1 mM EDTA, 1 mM EGTA, 2 mM PMSF, and leupeptin, aprotinin, and pepstatin at 1 μg/ml each), centrifuged (16,000 × g for 10 min at 4°C) and clarified lysates were assayed for total protein content (Bio-Rad, Cambridge MA). IL-1β or CX_3_CL1 concentration was determined in brain homogenates and plasma using commercial ELISA kits (R&D Systems, Minneapolis, MN). Absorbance (450 nm) was determined using a Bio-Tek synergy HT microplate reader (Bio-Tek Instruments, VT). Assays were sensitive to 1.5 pg/ml for IL-1β and 0.20 ng/ml for CX_3_CL1. For both assays, inter- and intra-assay coefficients of variation were less than 10%.

### Analysis of microglial gene expression and flow cytometry

Microglia were isolated from whole brain homogenates as previously described [5,34]. Brains were homogenized in Hank's Balanced Salt Solution (HBSS, pH 7.4) and centrifuged. Microglia were isolated using discontinuous Percoll density gradient (GE-healthcare, Uppsala, Sweden). The population that was isolated was found to be >90 pure microglia based on CD11b^+^/CD45^low ^phenotype.

Quantitative RT-PCR was performed using the Applied Biosystems (Foster, CA) Assay-on-Demand Gene Expression protocol as previously described [33]. In brief, RNA was isolated using the RNeasy plus mini kit (Qiagen, CA) and reverse transcribed to cDNA using an RT RETROscript kit (Ambion, TX). The target cDNA (e.g., CD11b, IL-1β, IDO, KMO TNFα, CD14 and TLR2) and a reference cDNA (glyceraldehyde-3-phosphate dehydrogenase) were amplified simultaneously. Data were analyzed using the comparative threshold cycle (Ct) method and results are expressed as fold difference from control.

Analysis of microglial surface antigens by flow cytometry was performed as previously described [34,35]. In brief, Fc receptors were blocked and microglia were incubated with anti CD14 and CD45 antibodies (eBioscience, CA). Antigen expression was determined using a Becton-Dickinson FACSCaliber four color Cytometer. Ten thousand events were recorded. For each antibody, gating was determined based on appropriate negative isotype stained controls. Flow data were analyzed using FlowJo software (Tree Star, CA).

### Neurochemistry

Tryptophan (TRP) and kynurenine (KYN) were determined as previously described [22]. In brief, plasma samples were deproteinated with 10% sulfosalicylic acid (5:1, v:v). Brain tissue was mechanically homogenized in 0.1N HClO_4 _+ 25 μM ascorbate. Samples were precipitated on ice for 30 min and centrifuged at 12,000 × g for 10 minutes at 4°C. The resulting supernatants were loaded into a Costar Spin-X centrifuge tube filter (0.22 μM Nylon Part #8169 Corning Incorporated) and centrifuged at 12,000 × g for 6 minutes at 4°C. Levels of tryptophan (TRP) and kynurenine (KYN) in the plasma and brain were determined using an ESA Coulochem III detector with a 5041 Enhanced Analytical cell containing a glassy carbon electrode (+600 mV). Samples were separated using a Mobile phase of 75 mM NaH_2_PO_4_, 25 μM EDTA-disodium salt, and 100 μl/L triethylamine in acetonitrile:water (6:94 v:v; pH 4.6).

Monoamines and metabolites were analyzed in whole brain as previously described [36]. Dopamaine (DA), homovanillic acid (HVA), and 3,4 Dihydroxyphenylacetic acid (DOPAC), serotonin (5-hydroxytryptophan; 5-HT) 5-hydroxyindoleacetic acid (5-HIAA), were analyzed at a potential of +320 mV, and the mobile phase (pH = 3.0) consisted of 75 mM NaH_2_PO_4_, 25 μM EDTA (disodium salt), 1.7 mM octanesulfonic acid, and 100 μl/L triethylamine in acetonitrile:water (6:94 v:v). The chromatograms were integrated and quantified using ESA EZ Chrom SI software (ESA Inc., Chelmsford, MA).

### Immunohistochemistry and digital image analysis

Mice were deeply anesthetized and transcardially perfused with sterile PBS followed by 4% formaldehyde. Brains were post-fixed in 4% formaldehyde for 24 h and then cryoprotected in 20% sucrose for an additional 24 h. Preserved brains were frozen using dry-ice cooled isopentane (-165°C) and then sectioned (20 μm) using a Microm HM550 cryostat. The brain sections were identified by reference markers in accordance with the stereotaxic mouse brain atlas [37]. Sections were mounted on microscope slides and stored at -20°C before staining procedures. Iba1 staining was performed as previously described [38]. In brief, sections were blocked and then incubated with rabbit anti-mouse Iba-1 antibody (Wako Pure Chemical Industries, Ltd., VA) overnight at 4°C. Next, HRP-conjugated goat anti-rabbit secondary antibody for 1 h at room temperature and staining was developed using the Vector VIP kit (Vector laboratories, CA).

For fluorojade-C staining, sections were prepared as described above and then incubated with 1% NaOH in ethanol and rinsed with 70% ethanol. Sections were then incubated with 0.06% potassium permanganate solution before incubation with 0.0001% fluorojade-C in 0.1% acetic acid. Slides were then washed with water and air dried, cleared with histoclear, and mounted with permount.

Fluorescent and brightfield images were visualized using an epifluorescent Leica DM5000B microscope. Images were captured using a Leica DFC300 FX camera and imaging software. To quantify the phenotypic changes of microglia, digital image analysis (DIA) of Iba1 staining was performed [39] in the hippocampus (-.1.7 to -2.3 mm bregma), basolateral amygdala (-1.7 to -2.06 mm bregma), and prefrontal cortex (+2.34 to +1.98 mm bregma) for each sample. To determine Iba1 phenotype in the entire hippocampus, at least three representative images were taken at 20× magnification in the dentate gyrus, CA1, and CA3 region of the hippocampus. For the basolateral amygdala and prefrontal cortex, at least 6 representative pictures were analyzed. A threshold for positive staining was determined for each image that included all cell bodies and processes, but excluded background staining. Thresholded targets were analyzed using densitometry and ImageJ software. Results were reported as the average percent area in the positive threshold for all representative pictures.

### Statistical Analysis

To ensure a normal distribution, data were subjected to Shapiro-Wilk test using Statistical Analysis Systems (SAS) software (Cary, NC). Observations greater than 3 interquartile ranges from the first and third quartile were considered outliers and were excluded in the subsequent analysis. To determine significant main effects and interactions between main factors, data were analyzed using one- (Genotype, Treatment, Time), two- (Genotype × Treatment, Treatment × Time) or three- (Genotype × Treatment ×Time) way ANOVA using the General Linear Model procedures of SAS. When appropriate, differences between treatment means were evaluated by an *F*-protected *t*-test using the Least-Significant Difference procedure of SAS. All data are expressed as treatment means ± standard error of the mean (SEM).

## Results

### Exaggerated social withdrawal after LPS injection in CX_3_CR1^-/- ^mice

To begin to explore the hypothesis that CX_3_CR1-deficient mice have significant behavioral complications after transient activation of the innate immune system, motivation to engage in social exploratory behavior was assessed. In this experiment, behavior was determined in CX_3_CR1^+/- ^(Control) and CX_3_CR1^-/- ^(KO) mice prior to i.p. injection of LPS and again 4, 8, 12, and 24 h after treatment. LPS injection exaggerated social withdrawal in CX_3_CR1^-/- ^mice compared to control mice (genotype × LPS interaction, F(1,149) = 16.35, *P *< 0.0001). This exaggerated social withdrawal in CX_3_CR1^-/- ^mice after LPS was dependent on time (tendency for genotype × LPS × time interaction, F(4,149) = 2.32, *P *= 0.06). For example, CX_3_CR1^-/- ^mice had an 87.2 ± 5.2% reduction in social exploration at 8 h after LPS while control mice had only a 52.7 ± 10.5% reduction (*P *< 0.001). Furthermore, control mice recovered to baseline social behavior by 24 h after injection, but CX_3_CR1^-/- ^mice had not recovered (*P *< 0.001). Taken together, these data indicate that LPS caused a prolonged reduction in social exploratory behavior in CX_3_CR1^-/- ^mice compared to CX_3_CR1^+/- ^controls.

**Figure 1:**
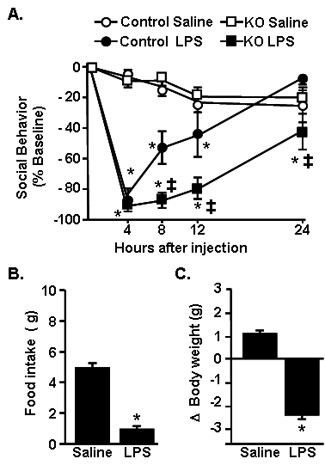
**LPS injection caused exaggerated social withdrawal in CX_3_CR1^-/- ^mice**. A) CX_3_CR1^+/- ^(Control) or CX_3_CR1^-/- ^(KO) mice were injected i.p. with saline or LPS (0.5 mg/kg) and social exploratory behavior was measured before LPS injection and again 4, 8, 12 and 24 h later. Graph represents the mean ± SEM (n = 10). Means with * are significantly different (*P *< 0.05) from respective saline-treated controls and means with ‡ are significantly different from *Control LPS*. B) Body weight and C) food intake were determined before i.p. LPS injection and again 4, 8, 12, and 24 h later. Because there was no significant effect of genotype, data were collapsed to show the main effect of LPS. Bars represent the mean ± SEM (n = 10). Means with * are significantly different (*P *< 0.05) from saline controls.

Food intake and change in body weight were also determined over the 24-h period after LPS injection. Because there was not a significant interaction between genotype and LPS, these data were collapsed to show the main effect of LPS. LPS injection caused a significant reduction in food intake (Figure 1B; F(1,29) = 118.9, *P *< 0.0001) and body weight (Figure 1C; F(1,29) = 199.5, *P *< 0.0001) that did not differ according to genotype.

### Higher levels of IL-1β protein in the cortex and plasma in CX_3_CR1^-/- ^mice after LPS injection

**Figure 2:**
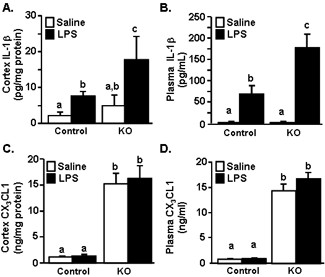
**LPS injection caused an exaggerated increase in IL-1β in the brain and plasma of CX_3_CR1^-/- ^mice**. CX_3_CR1^+/- ^(Control) or CX_3_CR1^-/- ^(KO) mice were injected i.p. with saline or LPS (0.5 mg/kg) and IL-1β protein levels determined in the A) cortex and B) plasma collected 4 h after injection. Bars represent the mean ± SEM. Means with different letters (a, b, or c) are significantly different (*P *< 0.05) from each other. In a related study, mice were treated as above and CX_3_CL1 protein levels were determined in the C) cortex and D) plasma collected 4 h after injection. Bars represent the mean ± SEM. Means with different letters (a, b, or c) are significantly different (*P *< 0.05) from each other

The pro-inflammatory cytokine, interleukin (IL)-1β is a key cytokine in the induction of sickness behavior [40,41]. Therefore, IL-1β protein levels were determined in the brain and plasma of control and CX_3_CR1^-/- ^mice 4 h after LPS injection. Figure 2A shows LPS injection increased IL-1β protein in the cortex (F(1,17) = 6.95, *P *< 0.05) and that IL-1β protein levels were highest in the cortex of CX_3_CR1^-/- ^mice compared to all other treatment groups (*P *< 0.05). Plasma levels of IL-1β were also increased by LPS injection (F(1,17) = 43.92, *P *< 0.0001) and these levels were higher in CX_3_CR1^-/- ^mice than in all other groups (Figure 2B; genotype × LPS interaction, F(1,17) = 8.38, *P *< 0.05).

A previous study showed that CX_3_CR1^-/- ^mice have significantly higher levels of CX_3_CL1 in the brain and plasma than control mice [42]. It is not known if CX_3_CL1 protein levels are changed in CX_3_CR1^-/- ^mice during a transient sickness response induced by LPS. Furthermore, increases in CX_3_CL1 protein in the brain after LPS injection could be involved in recruiting peripheral immune cells to the brain [43-45]. Therefore, total CX_3_CL1 protein was determined in the cortex and plasma. Levels of CX_3_CL1 were higher in the plasma and cortex of CX_3_CR1^-/- ^mice (Figure 2C &2D, main effect of genotype, F(1,15) = 230.82, *P *< 0.0001 and F(1,15) = 57.12, *P *< 0.0001, respectively). Consistent with a previous study using adult (3-4 mo) and aged (18-24 mo) Balb/c mice [5], CX_3_CL1 levels were not increased by LPS injection.

### Increased microglial mRNA expression of IL-1β, IDO and KMO in CX_3_CR1^-/- ^mice 4 h after LPS injection

**Figure 3:**
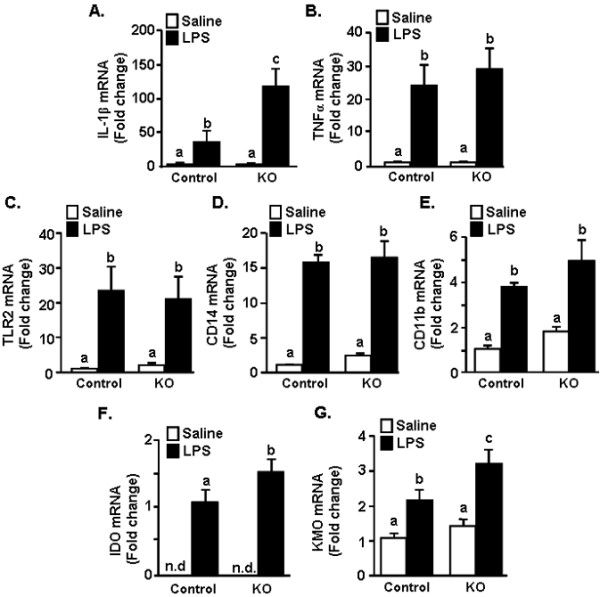
**Amplified IL-1β IDO and KMO mRNA induction in microglia of CX_3_CR1^-/- ^mice following peripheral LPS injection**. CX_3_CR1^+/- ^(Control) or CX_3_CR1^-/- ^(KO) mice were injected i.p. with saline or LPS (0.5 mg/kg) and A) IL-1β, B) TNFα, C) TLR2, D) CD14, E) CD11b, F) IDO and G) KMO mRNA levels determined from enriched microglia isolated 4 h after LPS injection. Bars represent the mean ± SEM. n.d. = values were non-detectable. Means with different letters (a, b, or c) are significantly different (*P *< 0.05) from each other.

Because IL-1β levels were highest in the cortex of CX_3_CR1^-/- ^mice after LPS injection, the contribution of microglia to these exaggerated IL-1β levels was assessed. In this experiment, adult CX_3_CR1^+/- ^and CX_3_CR1^-/- ^mice were injected i.p. with LPS and mRNA levels of several inflammatory-related genes, IL-1β, TNFα, TLR2, CD14, and CD11b were determined in enriched microglia collected 4 h later. Figure 3A shows that IL-1β, TNFα, TLR2, CD14, and CD11b mRNA expression in enriched microglia was increased 4 h after peripheral LPS injection (Figure 3A-E; main effect of LPS, *P *< 0.01 for each) and this LPS-induced increase was more marked in microglia of CX_3_CR1^-/- ^mice for IL-1β, only (genotype × LPS interaction, F(1,31) = 3.24, *P *= 0.08).

The mRNA levels of IDO and KMO, two genes associated with metabolism of tryptophan [4] were also determined. IDO activity is an important mediating factor in depression-like behavior after LPS injection in C57B/6 mice [20]. Furthermore, downstream processing of KYN by KMO into neuroactive metabolites is linked with depression in human patients undergoing IFN-alpha stimulation [46]. IDO mRNA was higher in microglia of LPS-treated CX_3_CR1^-/- ^mice than microglia of LPS-treated control mice (Figure 3F; F(1,19) = 2.9, P < 0.05). KMO mRNA levels were increased by LPS (F(1,38) = 20.9, *P *< 0.0001) and genotype (F(1,38) = 4.75, *P *< 0.05) with the highest levels in the microglia of CX_3_CR1^-/- ^mice that received LPS (*P *< 0.05). Collectively, these data indicate that microglia of CX_3_CR1-deficient mice have exaggerated RNA induction of IL-1β, IDO, and KMO compared to the CX_3_CR1^+/- ^control mice 4 h after LPS injection.

### Prolonged microglial expression of IL-1β in CX_3_CR1^-/- ^mice 24 h after LPS injection

**Figure 4:**
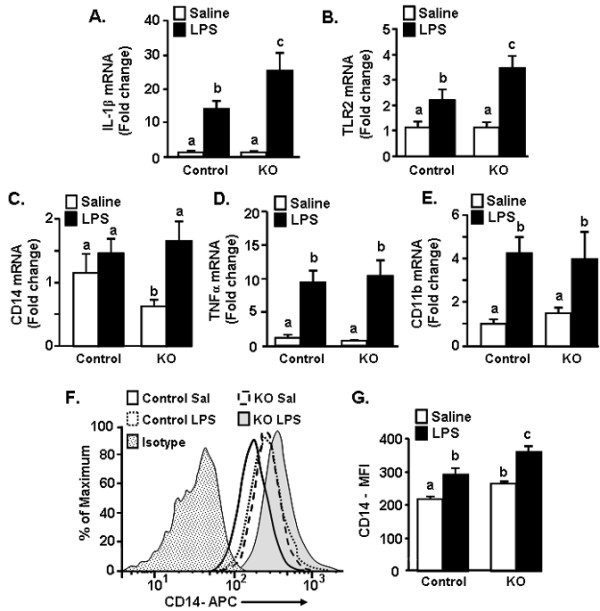
**Prolonged IL-1β, TLR2 and CD14 expression in microglia of CX_3_CR1^-/- ^mice following peripheral LPS injection**. CX_3_CR1^+/- ^(Control) or CX_3_CR1^-/- ^(KO) mice were injected i.p. with saline or LPS (0.5 mg/kg) and A) IL-1β, B) TNFα, C) TLR2, D) CD14 mRNA and E) CD11b levels were determined in enriched microglia isolated 24 h after LPS injection. Bars represent the mean ± SEM. Means with different letters (a, b, or c) are significantly different (*P *< 0.05) from each other. In a related study, control or KO mice were injected i.p. with saline or LPS and surface expression of CD14 protein on microglia was determined. F) Representative histograms of CD14 expression on microglia following experimental treatments. G) Mean fluorescence Intensity (M.F.I.) of CD14 expression on microglia (GFP^+^/CD45^low^) following experimental treatments. Bars represent the mean ± SEM (n = 6). Means with different letters (a, b, or c) are significantly different (*P *< 0.05) from each other.

The key issue with a deficiency in CX_3_CR1 surface expression may be associated with the duration that the microglia are actively producing cytokines [47]. Therefore, several inflammatory-related genes (IL-1β, TNFα, TLR2, CD14, and CD11b) and TRP metabolism-related genes (IDO and KMO) were determined 24 h after LPS injection. Figure 4A shows that IL-1β mRNA levels were still significantly elevated in microglia of CX_3_CR1^-/- ^mice compared to controls (genotype × LPS interaction, F(1,27) = 10.06, *P *< 0.01). TLR2 tended to be significantly elevated in microglia of CX_3_CR1^-/- ^mice compared to microglia of control mice (Figure 4B; genotype × LPS interaction, F(1,28) = 4.35, *P *= 0.09). CD14 mRNA was induced 24 h after LPS in CX_3_CR1^-/- ^(main effect of LPS, F(1,15) = 5.7, *P *< 0.05), but was unchanged in control mice after LPS (Figure 4C). Similar to the mRNA results at 4 h after LPS (Figure 3), TNFα and CD11b mRNA levels were increased by LPS, but these increases were independent of the CX_3_CR1 expression (Figure 4B&4D). LPS-induced increases in the mRNA levels of IDO and KMO in microglia were not detected at this 24 h time point (data not shown).

Because protein expression may not always coincide with mRNA transcription, a subset of mice were injected with saline or LPS and CD14 protein expression was determined on GFP^+^/CD45^low ^microglia 24 h later. Representative histograms of CD14 staining of microglia are shown in Figure 4F. LPS increased surface expression of CD14 on microglia (main effect of LPS, F(1,21) = 17.83, *P *< 0.001), and CD14 expression was the highest on microglia from LPS treated CX_3_CR1^-/- ^mice compared to all other treatment groups (*P *< 0.05). Taken together these data indicate that a specific subset of LPS-induced inflammatory markers are elevated longer in microglia of CX_3_CR1^-/- ^mice than in control mice.

### Increased turnover of TRP in the brain and plasma after LPS

**Figure 5:**
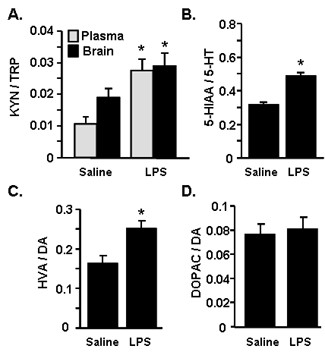
**LPS injection caused increased turnover of TRP, 5-HT, and DA**. CX_3_CR1^+/- ^(Control) or CX_3_CR1^-/- ^(KO) mice were injected i.p. with saline or LPS (0.5 mg/kg). Because there was no significant effect of genotype, data were collapsed to show the main effect of LPS. A) The ratio of KYN to TRP was determined in the brain and plasma 24 h later. B) The ratio of 5-HIAA to 5-HT, C) the ratio of HVA to DA, D) the ratio of DOPAC to DA were determined in the brain 24 h later. Bars represent the mean ± SEM. Means with * are significantly different (*P *< 0.05) from saline controls.

IDO is a key pathway in metabolism of TRP into KYN, and is involved in the induction of inflammatory-mediated depression in rodent models. Thus, the ratio of KYN to TRP is an accepted measure of IDO enzymatic activity [20,22]. Because there was an initial amplified induction of IDO in CX_3_CR1^-/- ^mice (Figure 3F&3G), KYN and TRP levels were determined in plasma and brain of control and CX_3_CR1^-/- ^mice 24 h after LPS injection. Although LPS injection significantly increased the ratio of KYN to TRP, there was not a significant interaction between genotype and LPS at this time point. For this reason, the data were collapsed to show the main effect of LPS treatment. Figure 5A shows that the ratio of KYN/TRP in the brain tended to be higher in LPS-injected mice compared to saline injected mice (F(1,29) = 4.02, *P *= 0.06). LPS also significantly increased the KYN/TRP ratio in the plasma (Figure 5A; F(1,29) = 13.6, *P *< 0.001).

Because proinflammatory cytokines directly alter the levels of monoamines and their metabolites [48], levels of serotonin (5-HT), 5-hydroxyindoleacetic acid (5-HIAA), dopamine (DA), homovanillic acid (HVA) and 3,4 Dihydroxyphenylacetic acid (DOPAC), were determined in the brain 24 h after LPS injection. Figure 5B shows that LPS injection markedly increased the ratio of 5-HIAA to 5-HT (F(1,31) = 41.48, *P *< 0.0001). LPS also increased the ratio of DA to HVA (Figure 5C; F(1,31) = 9.96, *P *< 0.01), but did not increase the ratio of DOPAC to DA (Figure 5D). In all cases, the increased ratio was related to an increase in the concentration of the metabolite (data not shown). Taken together these data indicate that there is increased metabolism of TRP, 5-HT and DA in the plasma and brain 24 h after LPS injection that is independent of CX_3_CR1 expression.

### Prolonged depression-like behavior detected only in CX_3_CR1^-/- ^mice after LPS

**Figure 6:**
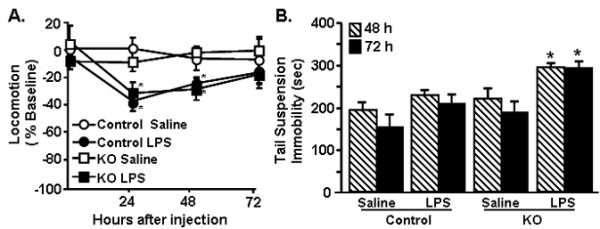
**Depression-like behavior was prolonged in CX_3_CR1^-/- ^mice following peripheral LPS injection**. CX_3_CR1^+/- ^(Control) or CX_3_CR1^-/- ^(KO) mice were injected i.p. with saline or LPS (0.5 mg/kg) and A) locomotor activity was determined 24, 48, and 72 h later. Bars represent the mean ± SEM (n = 10-12). Means with * are significantly different (*P *< 0.05) from LPS-treated group. B) Mice were treated as above and resignation was determined in the tail suspension test B) 48 (n = 16) and 72 h (n = 10) after injections. Bars represent the mean ± SEM. Means with * are significantly different (*P *< 0.05) from all other treatments.

We have previously reported that peripheral stimulation of the innate immune system with LPS in aged BALB/c mice (22-24 mo) causes prolonged sickness [33] and depression-like behaviors [19]. These behaviors are coupled with exaggerated microglial activation [35] and impaired CX_3_CR1-mediated regulation of microglia [5]. Based on these data, we next sought to determine the degree to which the depression-like behavior was evident in CX_3_CR1^+/- ^and CX_3_CR1^-/- ^mice after LPS injection. Depression-like behavior was assessed using the tail suspension test (TST), which is an assessment of motivation and resignation behavior in mice [19,49]. In this experiment, mice were injected with LPS and locomotor activity and resignation behavior were determined. Figure 6A shows that LPS caused a time-dependent reduction in locomotor activity (LPS × time interaction, F(3,152) = 2.84, *P *< 0.05) that was independent of genotype. All LPS-treated mice returned to baseline locomotor activity by 72 h after injection. Therefore, behavior was determined at 48 and 72 h. These two time points correspond with a time when mice were recovering from LPS-induced changes in locomotor activity (i.e., 48 h) and a time when they had fully recovered (i.e., 72 h). Figure 6B shows that immobility was increased after LPS (F(1,95) = 21.68, *P *< 0.0001) and that this increased resignation was only evident in LPS-treated CX_3_CR1^-/- ^mice (genotype × LPS interaction, F(1,95), 4.36, *P *< 0.05). These data indicate that LPS injection prolonged depression-like behavior in only CX_3_CR1^-/- ^mice.

### Activated microglial morphology is prolonged in CX_3_CR1^-/- ^mice 72 h after LPS

**Figure 7:**
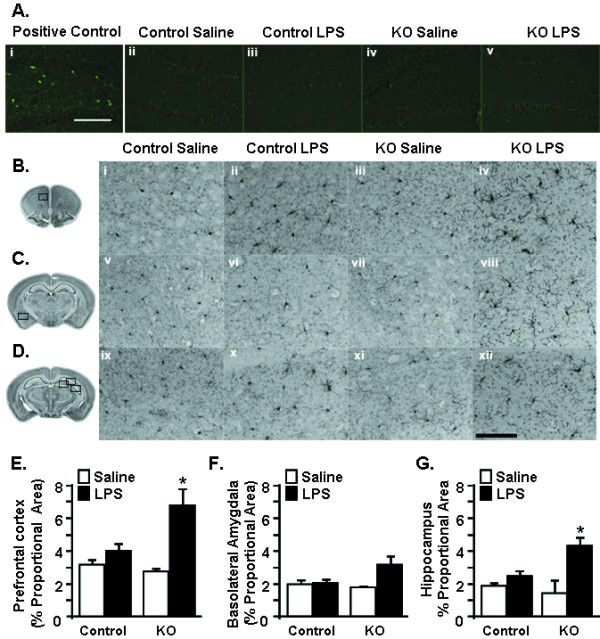
**Persistent activated morphology of microglia from CX_3_CR1^-/- ^mice following peripheral LPS injection**. CX_3_CR1^+/- ^(Control) or CX_3_CR1^-/- ^(KO) mice were injected i.p. with saline or LPS (0.5 mg/kg) and A) fluorojade-C staining was determined 72 h later. i) positive control, ii-v) representative pictures of Fluorojade-C staining in the hippocampus for each group. (B-D) Representative imagines of Iba-1 staining in the B) prefrontal cortex (PFC) C) basolateral amygdala (BLA), and D) CA1 region of hippocampus (HP). The black boxes over the brain slice images to the left indicates the region in which images were collected. To evaluate the entire hippocampus (HP), images were collected from the dentate gyrus, CA1 and CA3 regions (see methods). Proportional area of Iba-1 staining in the E) prefrontal cortex (n = 4), F) basolateral amygdala (n = 3), and G) hippocampus (n = 3). Bars represent the mean ± SEM. Means with * are significantly different (*P *< 0.05) from all other treatments.

We next sought to determine the degree to which prolonged resignation was associated with neuronal damage and microglia activation. In these experiments, CX_3_CR1^+/- ^and CX_3_CR1^-/- ^mice were injected i.p. with LPS and brains were collected 72 h after injection. To assess neuronal damage, the presence of fluorojade-c positive cells was determined. Brains of C57Bl/6 mice subjected to pilocarpine-induced status epilepticus were used as a positive control for neuronal damage [50]. (Figure 7A). While there was increased Fluorojade-c positive staining in the positive control in all of the examined brain regions increased Fluorojade-c staining was not detected in any of the other experimental groups (Figure 7A ii-v). Representative pictures in the dentate gyrus region of the hippocampus are shown. Neuronal damage was not found in the basolateral amygdala and prefrontal cortex in any experimental group (data not shown).

For the analysis of microglia activation, ionized calcium binding adapter molecule 1 (Iba1) staining was evaluated in the prefrontal cortex, basolateral amygdala, and hippocampus 72 h after injection. These regions were chosen because they are involved in mediating depression-like behavior in humans [51] and in mouse models [25]. Iba1 is an ideal marker for documenting microglia morphology in intact and pathological tissues [38]. Active microglia undergo hypertrophy and retract their processes [52]. Representative images of Iba1^+ ^microglia from each region are shown in Figure 7B-D. LPS-treated mice had larger cell bodies and thicker and condensed processes in the prefrontal cortex (Figure 7B) and the hippocampus (Figure 7D). In the prefrontal cortex of LPS-treated mice, digital image analysis [39] indicated that microglia had an increased proportional area of Iba1 immunoreactivity compared with microglia from saline-treated mice (Figure 7D; F(1,14) = 10.27, *P *< 0.001) and post hoc analysis revealed that Iba1 immunoreactivity in the prefrontal cortex was highest in LPS-treated CX_3_CR1^-/- ^mice (Figure 7D iv; *P *< 0.05). Moreover, in the hippocampus, LPS caused an increase in the in the proportional area for Iba1^+ ^microglia (F(1,12) = 5.98, *P *< 0.05) and was highest in CX_3_CR1^-/- ^mice compared to controls (Figure 7F xii, F(1,12) = 9.38, *P *< 0.05). There was no significant increase in Iba1 immunoreactivity in the basolateral amygdala 72 h after LPS (Figure 7E). Taken together these data indicate that peripheral injection of LPS causes morphological changes in microglia that are consistent with protracted activation and this occurs in the absence of increased neuronal death.

## Discussion

We have previously reported that stimulation of the peripheral innate immune system with LPS in aged BALB/c mice causes exaggerated microglial activation with amplified mRNA and intracellular protein expression of IL-1β [34] and prolonged reduction in microglial CX_3_CR1 expression when compared with microglia of adult mice [5]. The exaggerated neuroinflammation and microglial activation was paralleled by prolonged sickness behavior [33,34], impaired working memory [53], and protracted depression-like behavior [19]. These data support the hypothesis that impaired CX_3_CL1/CX_3_CR1 signalling in microglia is a key contributor to exaggerated behavioral impairments in response to LPS. For example, recovery of social exploratory behavior to baseline after LPS injection was delayed in CX_3_CR1^-/- ^mice compared to controls (Figure 1). Moreover, peripheral challenge with LPS caused exaggerated induction of IL-1β, IDO and KMO mRNA in the brain of CX_3_CR1^-/- ^mice (Figures 2). Also, several inflammatory markers (IL-1β, CD14, and TLR2) remained elevated in microglia of CX_3_CR1^-/- ^mice 24 h after LPS. Furthermore, depression-like behavior was detectable only in CX_3_CR1^-/- ^mice at 48 and 72 h after LPS injection (Figure 6). Last, the extended depression-like behavior corresponded with persistent morphological indices of microglial activation in the prefrontal cortex and hippocampus (Figure 7). Taken together, these data indicate that a deficiency in microglial regulation by CX_3_CR1 is permissive to prolonged neuroinflammation and behavioral complications after transient activation of the innate immune system.

It has been previously shown that CX_3_CR1^-/- ^mice have exaggerated microglial activation and neurotoxicity after repeated i.p. injections of LPS (4 daily injections; 20 ug) [10]. The results of the current study support these previous studies and show that a single acute injection of LPS (0.5 mg/kg) is sufficient to cause amplified (Figure 3) and prolonged microglial activation (Figures 4&7). With our injection protocol, however, the extended microglia activation was not associated with increased neuronal damage in the hippocampus (Figure 7A).

The present study also shows novel data that the loss of CX_3_CR1 was associated with pronounced social withdrawal (Figure 1A) and extended depression-like behavior (Figure 6B) after an acute injection of LPS. This protracted resignation behavior after LPS injection was evident only in the CX_3_CR1^-/- ^mice (Figure 6B) and was not associated with decreased locomotor activity (Figure 6A), exaggerated weight loss, or anorexia (Figures. 1B&1C). The findings of behavioral deficits in CX_3_CR1-deficient mice support our previous work with older BALB/c mice, in which the exaggerated sickness response after LPS injection [33] was also associated with prolonged depression-like behavior [19]. These findings are relevant because they suggest that a deficit in CX_3_CR1-mediated regulation of microglia is sufficient to cause prolonged sickness and depression-like behaviors after LPS injection even in the absence of the myriad inflammatory changes associated with aging.

The extended depression-like behavior at 72 h after LPS was associated with increased activated morphology of microglia in the prefrontal cortex and hippocampus (Figure 7B). Because the presence of increased iba1 immunoreactivity is indicative of activation [38], these data indicate that microglia are active longer in CX_3_CR1^-/- ^than control mice after LPS injection. In addition, the prefrontal cortex and the hippocampus are key brain regions associated with the regulation of mood and depression [54]. Other studies using restraint stress indicate that Iba1 immunoreactivity was increased in hippocampus, prefrontal cortex, and amygdala. This increased microglial activation in these areas was associated with anhedonia and depression-like behavior [25]. In the current study, significant increases of Iba1 immunoreactivity in the basolateral amygdala were not detected. This may be because CX_3_CL1 is highly expressed in the frontal cortex and hippocampus compared with the amygdala [7,55], indicating that CX_3_CL1 may have a greater role in regulating microglial activation in these areas. So, it is plausible that a deficiency of CX_3_CR1 could result in brain region-dependent differences in microglial activation after LPS.

Along with the presence of de-ramified microglia in LPS-treated CX_3_CR1^-/- ^mice, IL-1β protein levels were higher in the cortex of CX_3_CR1^-/- ^mice compared to controls (Figure 2A). There was also amplified and prolonged mRNA expression of IL-1β mRNA in microglia (Figure 3&4). Based on previous age comparisons with BALB/c mice, we expected that the majority of inflammatory genes examined would be higher in the CX_3_CR1^-/- ^mice. A more specific pattern of activation, however, was associated with CX_3_CR1-deficiency. For example, IL-1β, IDO, and KMO were highest in CX_3_CR1^-/- ^mice compared to all other groups 4 h after LPS (Figure 3A,F,G), whereas the LPS-induced increase of TNFα, TLR2, and CD14 expression was unaffected by a deficiency of CX_3_CR1 (Figure 3B-E). This pattern of activation may explain a key difference observed between aged mice and CX_3_CR1^-/- ^mice. Specifically, while aged mice show exaggerated anorexia and weight loss after LPS injection [33], CX_3_CR1^-/- ^mice do not (Figure 1). In previous age comparisons with BALB/c mice, TNFα was higher in the brain of aged mice after LPS compared with adult mice [56]. This is relevant because in a previous study, TNFα promoted anorexia in rodents while IL-1β promoted motivational changes, including increased social withdrawal behavior [57]. Thus, a specific increase of IL-1β in CX_3_CR1^-/- ^mice without a concomitant exaggeration of TNFα may enhance the behavioral effects of LPS without causing exaggerated anorexia and weight loss.

A deficiency of CX_3_CR1 on microglia also results in prolonged induction of select inflammatory markers after LPS injection. For instance, higher mRNA levels of TLR2 and IL-1β were still present in microglia of CX_3_CR1^-/- ^mice. Thus, CX_3_CR1-mediated regulation of IL-1β expression in microglia may play an important role in the resolution of brain inflammation and sickness behavior. In support of this notion, CX_3_CL1/CX_3_CR1 signalling activates the PI3K/Akt pathway in microglia [58] and this pathway is essential for fractalkine-signalling to attenuate IL-1β production in mixed glial cultures and microglial activation in aged rats [15]. In addition, LPS injection significantly decreases CX_3_CR1 surface expression on microglia; this downregulation is associated with increased IL-1β production and the promotion of sickness behavior. Recovery from the LPS-induced microglial activation and sickness behavior is dependent on restoration of normal CX_3_CR1 expression at 24 h after injection [5]. Taken together, we interpret these findings to suggest that a deficiency of CX_3_CR1 promotes a unique cytokine profile after LPS injection that specifically exaggerates LPS-induced mood and motivational changes.

Another important finding was that amplified induction of IDO and KMO was detected in the microglia of CX_3_CR1^-/- ^mice at 4 h after LPS injection. IDO metabolism of TRP into KYN is a key pathway involved in inflammatory-related depression in humans and in rodent models. Thus, the ratio of KYN/TRP is a proxy for IDO enzymatic activity. IDO activity is hypothesized to contribute to depression by generating neuroactive metabolites of kynurenine including 3-HK and QUIN. For instance, in clinical studies, depression is significantly higher in patients undergoing therapy with IFNα and this is associated with increased TRP and KYN levels in the plasma [59,60] and increased QUIN in the cerebrospinal fluid [46]. Therefore, we hypothesized that the increased depression-like behaviors in CX_3_CR1^-/- ^mice would be coupled with exaggerated turnover of TRP, along with changes in monoamine turnover. Consistent with previous studies using C57BL/6 mice [20], LPS caused an increased turnover of TRP, DA and 5-HT in the brain and TRP in the plasma (Figure 5). There was not, however, an interaction between LPS and CX_3_CR1 expression at 24 h. It is possible that the increased KYN and changes in monoamine turnover persisted for longer in the CX_3_CR1^-/- ^mice at 48 and 72 h after LPS. Because brains from these time points were analyzed for Iba1, this was not determined.

It is also possible that downstream metabolism of TRP through the KMO pathway is an important contributor to prolonged depression. Indeed, KMO activity is selectively increased in the rat brain after LPS injection [61]. A previous study indicated that IDO inhibition with 1-methyl tryptophan (1-MT) reversed LPS-induced depression independently of 5-HT turnover [20]. This same report indicates that peripheral administration of L-KYN in non-immune stimulated mice mimicked the depression-like effects of LPS [20]. Thus, although CX_3_CR1 deficiency did not affect KYN/TRP ratios, an exaggerated induction of KMO in microglia of CX_3_CR1^-/- ^mice after LPS (Figure 3) may result in greater accumulation of 3-HK and QUIN in the brain and contribute to the extended depression-like behavior in the TST.

It is important to note that CX_3_CL1/CX_3_CR1 signalling events are dependent on the nature of the inflammatory stimulus. For instance, experimental autoimmune encephalomyelitis (EAE) [44,62] and ischemia [43,63] are associated with an induction of CX_3_CL1 and an accumulation of CX_3_CR1-expressing cells. Therefore, neuronal or glial release of CX_3_CL1 may be a mechanism by which microglia and other CX_3_CR1^+ ^immune cells are recruited to damaged areas within the CNS. Notably, CX_3_CR1-deficient mice exhibit reduced leukocyte infiltration after ischemic injury that is associated with smaller infarcts and reduced inflammation after injury [43]. In contrast, CX_3_CR1-deficient mice were more susceptible to EAE-related death and haemorrhagic brain lesions [44]. The exaggerated EAE was associated with insufficient recruitment of NK cells into the brain of CX_3_CR1^-/- ^mice. A recent study demonstrated that CX_3_CR1^-/- ^mice show enhanced CX_3_CL1 expression in the plasma and brain [42]. In the present study, plasma and cortex levels of CX_3_CL1 where elevated in the CX_3_CR1-deficient mice, but were not affected by LPS (Figure 2C&2D). The lack of an LPS-induced increase of CX_3_CL1 argues against the idea that our LPS stimulus is associated with an influx of CX_3_CR1^+ ^cells that contribute to sickness and depressive behaviors. Furthermore, these data support the idea that it is the regulation of CX_3_CR1 on microglia, rather than LPS-induced changes in CX_3_CL1, that underlies neurobehavioral changes after LPS injection [5]. Therefore, in the context of an acute immune activation, our data support the idea that CX_3_CL1-CX_3_CR1 interactions function to regulate the activation of microglia separately from peripheral interactions.

## Conclusions

In conclusion, the present study provides novel evidence that impaired regulation of microglia through CX_3_CR1 has profound on subsequent neurobehavioral deficits after injection of LPS. These data along with our previously published reports support the hypothesis that the CX_3_CR1 is important regulator of microglia.

## Competing interests

The authors of this manuscript declare that there are no actual or potential conflicts of interest. The authors affirm that there are no financial, personal or other relationships with other people or organizations that have inappropriately influenced or biased their research.

## Authors' contributions

AWC was responsible for executing the research project, completion of statistical analysis, and writing of the manuscript. YH and JCO assisted technically with research and data analysis. KWK, RD, PGP contributed to the design of the experiments and assisted in editing the manuscript. JPG directed all aspects of this research project including the experimental design, completion of statistical analysis, and writing of the manuscript. All authors read and approved the final manuscript.
